# Metachronous Bilateral Isolated Adrenal Metastasis from Rectal Adenocarcinoma: A Case Report

**DOI:** 10.1155/2014/516403

**Published:** 2014-04-22

**Authors:** H. Jabir, N. Tawfiq, M. Moukhlissi, M. Akssim, A. Guensi, B. Kadiri, Z. Bouchbika, A. Taleb, N. Benchekroun, H. Jouhadi, S. Sahraoui, S. Zamiati, A. Benider

**Affiliations:** ^1^Mohammed VI Centre for the Treatment of Cancers, CHU Ibn Rochd, Casablanca, Morocco; ^2^Department of Anatomy and Pathological Cytology, CHU Ibn Rochd, Casablanca, Morocco; ^3^Department of Nuclear Medicine, CHU Ibn Rochd, Casablanca, Morocco; ^4^Department of Surgery, CHU Ibn Rochd, Casablanca, Morocco

## Abstract

We report a case of adrenal metastasis from colorectal cancer in a 54-year-old woman. Nine months after resection for advanced rectal carcinoma, a computed tomography scan revealed bilateral adrenal metastasis. The level of serum carcinoembryonic antigen was normal. A bilateral adrenalectomy was performed after chemotherapy. Histopathological examination showed adenocarcinoma, compatible with metastasis from the rectal cancer. Adrenal metastasis should be considered in the patients' follow-up for colorectal cancer.

## 1. Introduction

Adrenal metastasis is regarded as an indicator of systemic disease [1]. Non-small-cell lung cancer (NSCLC) is the most frequent tumor type followed by colorectal and renal cell carcinoma [2].

The isolated adrenal metastases from colorectal cancer are exceptional, especially when they are bilateral. We report a case of solitary bilateral adrenal metastasis from rectal carcinoma with a review of the relevant literature.

## 2. Case Report

A 54-year-old woman had a history of altered bowel habit, rectorragia, and weight loss for approximately 6 months. Rectoscopy showed a low rectal tumor. Microscopic examination confirmed rectal adenocarcinoma. There was not any evidence of metastatic disease.

Concurrent chemotherapy/radiotherapy of the pelvis with 6 MV photons of 45 Gy in 25 fractions over 5 weeks was performed. The woman had an abdominoperineal amputation 6 weeks later.

Microscopic examination did not find a residual tumor. The stage was YpT3N-M0 according to TNM 2009.

**Figure 1:**
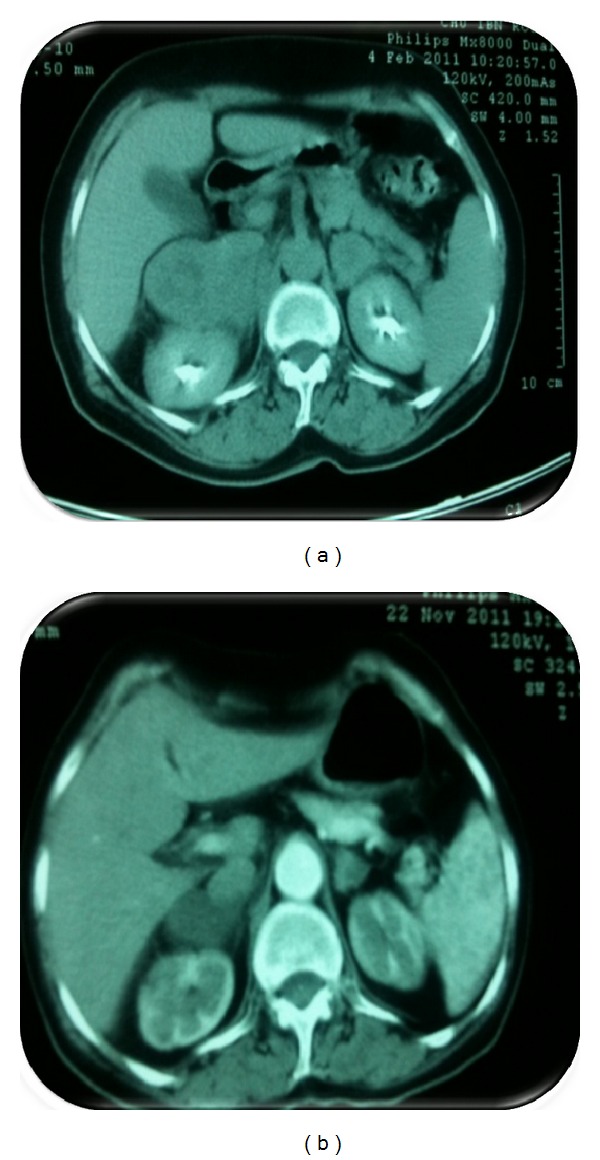
(a) Computed tomography of the abdomen shows a bilateral adrenal mass. (b) CT evaluation showed response of the adrenal metastases after chemotherapy.

Nine months after the surgery, thoracoabdominopelvic CT scan showed bilateral adrenal masses widely necrosed. These masses measured, respectively, 67 mm to the right and 59 mm to the left with no evidence of the disease elsewhere (Figure 1).

The serum carcinoembryonic antigen (CEA) levels were consistently within the normal range.

Systemic chemotherapy was initiated. The patient underwent 9 cycles (FOLFIRI-Avastin) with a good clinical and biological tolerance.

The CT scan evaluation showed a partial answer of 50% to the right and 66% to the left (Figure 1).

A right adrenalectomy was performed. The left adrenalectomy was not realized because of the hemorrhage during the dissection. Histology was consistent with the material difficult to typify.

**Figure 2:**
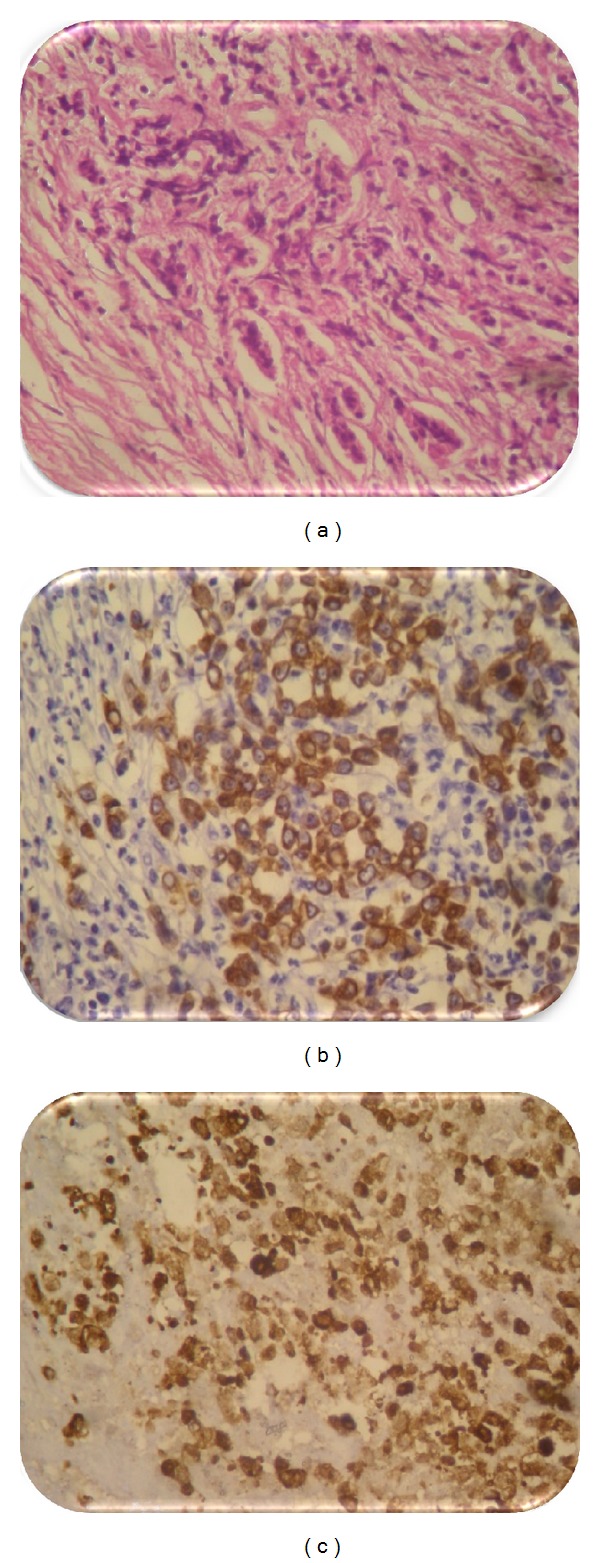
(a) Histopathological findings showed cells difficult to typify (40). (b) Immunohistochemistry of the piece showed intense expression of cytokeratin (AE1/AE3). (c) Expression of CK20 similar to the primary colorectal carcinoma.

Immunohistochemistry was positive for CK20, cytokeratin AE1/AE3, but negative for CK7, which is related to colorectal metastasis (Figure 2).

**Figure 3:**
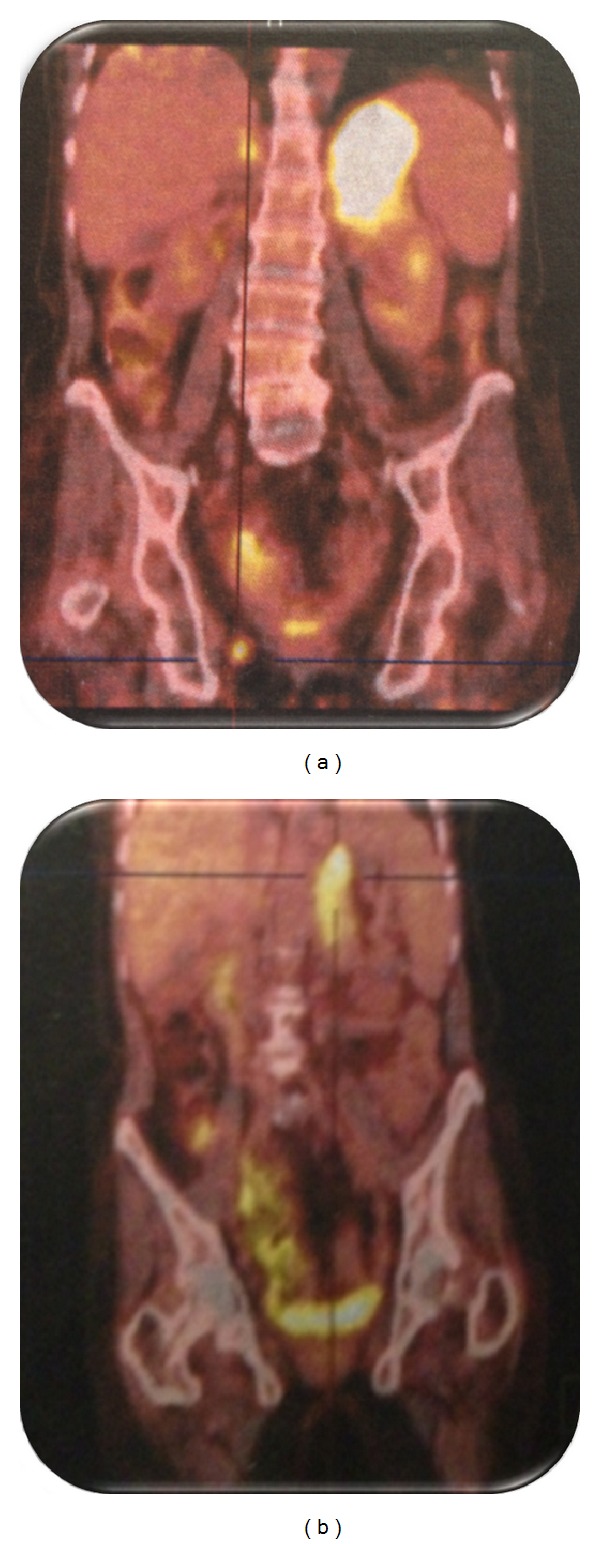
(a) PET scan showed suspicious hypermetabolic sites in the left suprarenal gland, lomboaortic nodes, liver, and the left kidney. (b) PET scan showed a remission estimated at 90% on the left suprarenal gland and the disappearance of the metastasis at the level of the other sites after chemotherapy.

After surgery, a PET scan showed hypermetabolic sites of malignancy in left suprarenal gland, lomboaortic nodes, liver, and left kidney (Figure 3).

Chemotherapy (FOLFIRI-Avastin) was taken back. Our patient received 6 cycles and she kept a good general health.

After the 6th cycle, PET scan showed a disappearance of the metastasis of lomboaortic nodes, liver, and left kidney and a decrease of the left adrenal gland (estimated at 90%) (Figure 3).

Excision of the left adrenal gland was done. Microscopic examination showed adenocarcinoma, compatible with metastasis from the rectal carcinoma.

Chemotherapy based on FOLFOX-Avastin is underway. The patient is still in good general condition.

## 3. Discussion

Adrenal metastasis is reported to be frequently found at autopsy [1]. They represent the 4th metastatic site after lung, liver, and bone [3]. The primitive cancers most often met are the lung, the colorectal, and renal cancers [2].

Adrenal metastasis from colorectal carcinoma is relatively rare with an incidence from 3.1% to 14.4% [1]. However, this incidence can be underestimated because an adrenal mass can be considered as a lomboaortic node [4].

Adrenal metastasis is regarded as an indicator of systemic disease [1]. A lot of paths exist, including systemic venous, portal venous, arterial, and lymphatic paths.

Low rectal has double vascularization by the lower mesenteric artery and intern iliac artery. His anatomical particularity could explain the fact that cancer cells can borrow the vena cava inferior directly towards the lung and then towards the general circulation and finally at the level of the suprarenal gland.

Katayama et al. [5] and Yasuhiro (Department of Surgical Oncology, Faculty of Medicine, The University of Tokyo, Tokyo, Japan) suggested that rectal metastasis can reach the adrenal gland via the lung.

It is exceptional to find bilateral isolated adrenal metastases from rectal cancer to a live patient. To our knowledge, only 2 cases of isolated bilateral adrenal metastases of the low rectal cancer were described in the literature until now [6, 7].

The imaging has a very important role in the diagnosis of the adrenal metastases. They used to be discovered by autopsies. In fact, because of improved imaging and diagnostic techniques, many adrenal metastases are now discovered on routine follow-up imaging [1].

The computed tomography is the reference examination. The objective is to make the difference of adrenal incidentaloma [8].

The adrenal metastasis is usually interpreted in the CT by a nodule of the order of 5 cm, spontaneously dense, with irregular limits, which takes a heterogeneous aspect after injection of contrast agent [6–9].

The CT of our patient noticed adrenal nodules exceeding 5 cm of size, heterogeneous, and asymmetric which are very in favor of the adrenal metastases.

The use of PET scan in staging and surveillance of the colorectal cancer allowed the detection and specifies adrenal metastases by a better characterization of the metabolic activity. It permits choosing an adapted treatment and avoiding a useless laparotomy in case of generalized metastases [10, 11].

The staging was realized at our patient by a thoracoabdominopelvien CT. The PET scan was not available in our establishment at the time of the diagnosis of the adrenal metastases.

Fine-needle aspiration biopsy of adrenal masses is the best means to confirm the diagnosis of metastasis. But there is a risk of high complications as the pneumothorax and the retroperitoneal hemorrhage due to the deep topography of suprarenal glands [12].

Fine-needle aspiration biopsy was not made to our patient because the CT characteristics and the story of the disease were sufficient to retain the diagnosis of the adrenal metastases.

Tumor marker CEA is generally raised during the diagnosis of metastases. It is considered as a good indicator of the presence of adrenal metastasis after surgery [13]. In our case, the ACE remained negative; it was never contributory for the diagnosis of the adrenal metastases.

Therapeutic approach of metastatic colorectal cancer underwent a change mattering during the last five years. The overall survival can reach at present more than 20 months due to the progress of the chemotherapy and targeted therapies [14].

There is a consensus for the treatment of the hepatic metastases of colorectal cancer [15] but no standard exists for the extrahepatic metastasis.

The surgical resection of the adrenal metastases remains controversial because they usually evoke a generalized disease.

The only series including 8 patients treated for isolated adrenal metastases of a colorectal carcinoma reports a median survival of 32 months [16].

Resection of the isolated adrenal metastases from colorectal cancers will be proposed for the patients whose appearance of the adrenal metastasis is up to 6 months because the surgery allows increasing the chances of the survival [5, 17].

We opted for surgery of adrenal metastasis for several reasons.

- The metastases were isolated.
- The patient has responded to the neoadjuvant chemotherapy.
- Resection of the isolated adrenal metastases provides better survival especially when it has developed after 6 months of the surgery [5, 17, 18].

There is no evidence of adjuvant chemotherapy for the adrenal metastases. Biochemotherapy (oxaliplatin-irinotecan) and biotherapy (bevacizumab-cetuximab) are recommended for adrenal metastasis in the optics of a secondary resectability.

## 4. Conclusion

Bilateral isolated adrenal metastases of a colorectal cancer are very rare. The prognosis is relatively good if metastases are detected and resected early. Because of their rarity, there are no, at the moment, randomized studies which support the efficiency of the surgery.
